# Cryptococcal Meningitis Revealing Late-Onset Combined Immunodeficiency After Two Decades of Recurrent Infections: A Case Report

**DOI:** 10.7759/cureus.112980

**Published:** 2026-07-19

**Authors:** Valeria J Martínez Evangelista, Sandra Munoz Plascencia, Juan C Cárdenas-Favela, Carlos A Correa Serrano

**Affiliations:** 1 Internal Medicine, Hospital Regional Dr. Valentín Gómez Farías, Zapopan, MEX; 2 Internal Medicine, Hospital General Zona No. 3 "Dr. Héctor González Guevara", Mazatlán, MEX; 3 Allergy and Clinical Immunology, Hospital General Zona No. 3 "Dr. Héctor González Guevara", Mazatlán, MEX; 4 Allergy and Immunology, Hospital Regional Dr. Valentín Gómez Farías, Zapopan, MEX

**Keywords:** cd4+ lymphocytopenia, cryptococcal meningitis, hypogammaglobulinemia, inborn errors of immunity, late-onset combined immunodeficiency

## Abstract

Adult-onset inborn errors of immunity pose a significant diagnostic challenge because of their non-specific clinical presentation and frequent delay in diagnosis. We report a case of a 53-year-old woman with a nearly two-decade history of recurrent infections whose clinical course culminated in cryptococcal meningitis, prompting a comprehensive immunologic evaluation. Following the systematic exclusion of secondary causes of immunodeficiency, immunologic testing revealed hypogammaglobulinemia and profound CD4+ T-cell lymphopenia (188 cells/µL), consistent with combined humoral and cellular immune dysfunction and supporting classification within the late-onset combined immunodeficiency (LOCID) phenotype. Following appropriate antifungal therapy, subsequent immunoglobulin replacement therapy and filgrastim were associated with sustained clinical improvement. This case highlights that cryptococcal meningitis in HIV-negative patients should prompt a systematic evaluation for an underlying primary immunodeficiency and underscores the importance of recognizing the LOCID phenotype as an uncommon but potentially treatable cause of opportunistic infections in adults.

## Introduction

Inborn errors of immunity (IEIs) comprise a heterogeneous group of genetically determined disorders that impair the development and function of the immune system. Although traditionally regarded as pediatric diseases, it is now well recognized that an increasing number of patients remain undiagnosed until adulthood because of slowly progressive phenotypes and non-specific clinical manifestations. The most recent International Union of Immunological Societies (IUIS) classification highlights the expanding spectrum of IEIs and emphasizes the need to consider these disorders in patients presenting with recurrent infections, opportunistic infections, or unusual immune-mediated manifestations [1,2].

Common variable immunodeficiency (CVID) is the most prevalent symptomatic primary immunodeficiency in adults and remains a diagnosis of exclusion. The International Consensus Document (ICON) recommends integrating clinical and immunologic findings to establish the diagnosis, while current guidelines emphasize the systematic exclusion of secondary causes of hypogammaglobulinemia before a primary immunodeficiency is considered [3,4]. Within the clinical spectrum of CVID, late-onset combined immunodeficiency (LOCID) is recognized as a severe clinical phenotype rather than a universally accepted distinct disease entity. It is characterized by profound CD4+ T-cell lymphopenia, increased susceptibility to opportunistic infections, and combined humoral and cellular immune dysfunction, reflecting a more aggressive clinical course than classical CVID [5]. Because universally accepted diagnostic criteria are lacking, recognition of LOCID continues to rely on comprehensive clinical assessment, immunologic evaluation, and systematic exclusion of secondary causes of immunodeficiency [6].

We report a case of a 53-year-old woman with a nearly two-decade diagnostic delay and a history of recurrent bacterial and viral infections whose clinical course culminated in cryptococcal meningitis, the sentinel event that prompted a comprehensive immunologic evaluation and ultimately supported classification within the LOCID phenotype. This case highlights the importance of considering IEIs in adults with opportunistic infections and underscores the need for heightened clinical suspicion among internists to facilitate timely diagnosis and early initiation of targeted therapy capable of improving clinical outcomes.

## Case presentation

A 53-year-old woman from Jalisco, Mexico, with a history of well-controlled hypertension receiving antihypertensive therapy and no known drug allergies, was referred to the internal medicine department. Her family history was notable for the death of her mother from an unspecified bone marrow disorder.

Retrospective review of her medical history revealed an unrecognized pattern of recurrent infections spanning nearly two decades. At 35 years of age, she experienced multiple episodes of otitis media and otitis externa requiring hospitalization and intravenous antibiotic therapy. At 43 years of age, she developed recurrent herpes zoster involving the trunk, followed by Ramsay Hunt syndrome. In 2015, she was diagnosed with malignant otitis externa, and three years later required hospitalization for community-acquired pneumonia, followed by a subsequent episode of viral pneumonia. Beginning in 2022, she reported intermittent diarrhea occurring approximately once per month, accompanied by unintentional weight fluctuations. Collectively, these recurrent infections evolved over many years without a unifying diagnosis before her current presentation.

In August 2024, she presented with a three-week history of progressively worsening headache, intermittent fever, nausea, vomiting, and blurred vision. Five days before admission, she developed progressive somnolence and recent memory impairment, prompting evaluation for infectious meningoencephalitis. Cerebrospinal fluid (CSF) analysis revealed clear fluid with lymphocytic pleocytosis (61 cells/µL; 92% lymphocytes), elevated protein (108 mg/dL), hypoglycorrhachia (CSF glucose: 32 mg/dL; serum glucose: 100 mg/dL; CSF-to-serum glucose ratio: 0.32), and an elevated lactate concentration (4.8 mmol/L). Opening pressure was not recorded. Cryptococcal antigen testing in both CSF and serum was positive, and CSF culture grew *Cryptococcus neoformans*, confirming cryptococcal meningitis.

She received induction therapy with intravenous liposomal amphotericin B (3 mg/kg/day) for two weeks, followed by fluconazole consolidation therapy. Her hospital course was complicated by healthcare-associated pneumonia with parapneumonic pleural effusion and acute hypoxemic respiratory failure requiring invasive mechanical ventilation for five days. Following antifungal and broad-spectrum antimicrobial therapy, she recovered clinically and was successfully weaned from ventilatory support.

Given the occurrence of cryptococcal meningitis in an HIV-negative adult, an underlying inborn error of immunity was strongly suspected. Immunologic evaluation was therefore performed after completion of induction therapy, while the patient was receiving fluconazole consolidation therapy and was clinically stable, minimizing the potential impact of acute infection on immunologic testing. HIV serology was negative, and there was no history of corticosteroid use, biologic therapy, or other immunosuppressive medications. Nutritional assessment, metabolic evaluation, and investigation for protein-losing disorders were unremarkable, excluding secondary causes of hypogammaglobulinemia.

Persistent anemia, leukopenia with lymphopenia, and neutropenia initially raised concern for an underlying primary hematologic disorder, including myelodysplastic syndrome or bone marrow failure. Bone marrow aspiration with flow cytometric immunophenotyping demonstrated marrow hypocellularity without evidence of lymphoproliferative disease or malignant infiltration. Conventional cytogenetic analysis revealed a normal bone marrow karyotype, effectively excluding an underlying hematologic malignancy as the cause of her clinical presentation (Table 1).

**Table 1:** Laboratory, immunologic, hematologic, microbiologic, and genetic findings obtained during the diagnostic evaluation of the patient. HPF: high-power field; HCV: hepatitis C virus; VDRL: Venereal Disease Research Laboratory; FANCM: Fanconi anemia complementation group M

Category	Parameter	Reference range	Patient result
Complete blood count (CBC)	Hemoglobin (g/dL)	12.0-16.0	10.0
	Hematocrit (%)	36-46	30.4
	Red blood cell count (×10⁶/µL)	3.8-5.2	3.32
	Mean corpuscular volume (MCV, fL)	80-100	91
	Mean corpuscular hemoglobin (MCH, pg)	27-33	28
	White blood cell count (/µL)	4,000-10,000	640
	Neutrophils (%)	50-70	46.8
	Absolute neutrophil count (/µL)	1,500-7,500	300
	Lymphocytes (%)	20-45	26.6
	Absolute lymphocyte count (/µL)	1,000-4,000	170
	Monocytes (/µL)	200-1,200	30
	Eosinophils (/µL)	0-500	20
	Basophils (/µL)	0-100	10
	Platelet count (/µL)	150,000-450,000	230,000
Glycemic status	Hemoglobin A1c (%)	<5.7	5.4
Thyroid function tests	Thyroid-stimulating hormone (TSH, μIU/mL)	0.4-4.5	2.0
	Free thyroxine (free T4, ng/dL)	0.8-1.8	1.1
	Free triiodothyronine (free T3, pg/mL)	2.3-4.2	2.8
Lipid profile	Total cholesterol (mg/dL)	<200	188
	LDL cholesterol (mg/dL)	<100	88
	HDL cholesterol (mg/dL)	>50	50
	Triglycerides (mg/dL)	<150	166
Liver function tests	Aspartate aminotransferase (AST, U/L)	10-40	19
	Alanine aminotransferase (ALT, U/L)	7-56	22.6
	Alkaline phosphatase (U/L)	44-147	242
	Gamma-glutamyl transferase (GGT, U/L)	9-36	36
	Total bilirubin (mg/dL)	0.2-1.2	0.85
	Direct bilirubin (mg/dL)	<0.3	0.36
	Albumin (g/dL)	3.5-5.0	3.6
	Total protein (g/dL)	6.4-8.3	6.6
Inflammatory markers	C-reactive protein (mg/L)	<5	3
	Erythrocyte sedimentation rate (mm/h)	<15	19
	Lactate dehydrogenase (LDH, U/L)	140-280	270
Urinalysis	Specific gravity	1.005-1.030	1.020
	pH	5.0-8.0	6.0
	Protein	Negative	Negative
	Glucose	Negative	Negative
	Blood	Negative	Negative
	Leukocytes	0-5/HPF	2/HPF
	Erythrocytes	0-2/HPF	0/HPF
	Nitrites	Negative	Negative
Immunologic evaluation	IgG (g/L)	7-16	4.0
	IgA (g/L)	0.7-4.0	1.0
	IgM (g/L)	0.4-2.3	0.2
	Complement C3 (g/L)	0.90-1.80	1.3
	Complement C4 (g/L)	0.10-0.40	0.11
	CD3+ T cells (/µL)	700-2,100	470
	CD4+ T cells (/µL)	500-1,500	188
	CD8+ T cells (/µL)	300-1,000	270
	CD4/CD8 ratio	1.0-3.5	0.69
Serum protein studies	Serum protein electrophoresis	Polyclonal pattern	Polyclonal pattern
	Serum immunofixation	Negative	Negative
	Kappa free light chains (mg/L)	3.3-19.4	6.9
	Lambda free light chains (mg/L)	5.7-26.3	7.8
	κ/λ ratio	0.26-1.65	0.88
Infectious disease serology	HIV	Negative	Negative
	HBsAg	Negative	Negative
	Total anti-HBc	Negative	Negative
	Anti-HCV	Negative	Negative
	VDRL	Negative	Negative
Tumor markers	Carcinoembryonic antigen (CEA, ng/mL)	<5.0	1.0 (negative)
	CA 19-9 (U/mL)	<37	3 (negative)
	CA-125 (U/mL)	<35	33 (negative)
	Alpha-fetoprotein (AFP, ng/mL)	<10	1.0 (negative)
Bone marrow evaluation	Bone marrow aspirate	Hypocellular	Hypocellular
	Bone marrow immunophenotyping	No lymphoproliferative disorder	No lymphoproliferative disorder
	Bone marrow karyotype	Normal (46, XX)	Normal (46, XX)
Genetic testing	Whole-exome sequencing	No pathogenic variant identified	FANCM c.1741C>T (p.Arg581Cys), variant of uncertain significance (VUS)

The patient's longstanding history of recurrent bacterial and viral infections, together with the absence of an identifiable secondary cause of immunodeficiency, strongly suggested an adult-onset inborn error of immunity. Consistent with this clinical impression, she fulfilled multiple Jeffrey Modell Foundation warning signs for primary immunodeficiency in adults, including more than two new ear infections within one year, pneumonia occurring in more than one year, recurrent viral infections (herpes zoster), and the repeated need for intravenous antimicrobial therapy, thereby further supporting the indication for a comprehensive immunologic evaluation. Although the patient also reported chronic diarrhea, the absence of documented weight loss meant that this manifestation was not considered sufficient to fulfill the corresponding Jeffrey Modell criterion.

A comprehensive immunologic evaluation was subsequently performed, including serum immunoglobulin quantification, complement testing, and lymphocyte subset analysis. Serum IgG was markedly reduced at 4 g/L, whereas IgA was measured at 1 g/L. Complement fractions (C3 and C4) were within normal limits. Immunophenotyping revealed profound CD4+ T-cell lymphopenia, with an absolute CD4+ T-cell count of 188 cells/µL. Overall, the immunologic profile was consistent with combined humoral and cellular immune dysfunction rather than an isolated antibody deficiency. Preserved IgA levels and normal complement fractions excluded selective IgA deficiency and complement deficiencies as alternative explanations for the patient's clinical presentation.

To further investigate the underlying etiology, whole-exome sequencing together with a targeted inborn errors of immunity gene panel was performed. Genetic analysis identified a variant of uncertain significance (VUS) in FANCM (c.1741C>T; p.Arg581Cys). Following multidisciplinary review by the hematology and clinical immunology teams, the variant was considered insufficient to account for the patient's immunologic phenotype because it represented a non-truncating missense variant without functional evidence supporting pathogenicity. Although assessment of vaccine-specific antibody responses and functional lymphocyte assays would have provided additional functional characterization, these investigations were not available at our institution.

Accordingly, the patient was classified as having a clinical phenotype most consistent with late-onset combined immunodeficiency (LOCID). This classification was based on the combination of longstanding recurrent bacterial and opportunistic infections, profound CD4+ T-cell lymphopenia (188 cells/µL), hypogammaglobulinemia, systematic exclusion of secondary causes of immunodeficiency, and multidisciplinary interpretation of the clinical, immunologic, and genetic findings by the hematology and clinical immunology teams.

Intravenous immunoglobulin (IVIG) replacement therapy was initiated with a loading dose of 0.6 g/kg (42 g), together with filgrastim 300 μg once daily for five consecutive days. The patient demonstrated a marked clinical response, with complete resolution of the acute infection and recovery of neutrophil counts to within the reference range. At the time of hospital discharge, there was no clinical or microbiological evidence of ongoing infection, and she was scheduled for multidisciplinary outpatient follow-up with the internal medicine, hematology, and clinical immunology services.

Maintenance intravenous immunoglobulin (IVIG) therapy was continued at 0.4 g/kg (28 g) every four weeks. Over 12 months of follow-up, serial complete blood counts demonstrated sustained normalization of neutrophil counts. Although repeat immunologic evaluation, including CD4+ T-cell counts, B-cell and natural killer-cell subsets, and functional immune studies, was not available, the patient remained free of recurrent opportunistic infections and infection-related hospitalizations. During this period, she experienced only two uncomplicated urinary tract infections, both successfully treated in the outpatient setting. Overall, her clinical course was characterized by sustained hematologic recovery, clinical stability, and a marked reduction in infectious complications following appropriate antifungal therapy and subsequent immunoglobulin replacement, further supporting classification within the proposed LOCID phenotype.

## Discussion

Delayed recognition remains one of the greatest challenges in diagnosing adult-onset inborn errors of immunity (IEIs). Our patient experienced recurrent bacterial and viral infections for nearly two decades before developing cryptococcal meningitis, the sentinel event that prompted comprehensive immunologic evaluation. The current International Union of Immunological Societies (IUIS) classification recognizes that many IEIs remain undiagnosed until adulthood because of slowly progressive phenotypes and non-specific manifestations, leading many patients to be evaluated initially by internists rather than immunologists [1,2,7].

The diagnostic approach followed current international recommendations. The International Consensus Document (ICON) emphasizes that IEIs should be diagnosed through integration of clinical and immunologic findings after exclusion of secondary causes of hypogammaglobulinemia [3]. HIV infection, pharmacologic immunosuppression, metabolic disorders, protein-losing conditions, and hematologic malignancies were systematically excluded. Although persistent cytopenias and bone marrow hypocellularity initially raised concern for a primary marrow disorder, the absence of clonal or cytogenetic abnormalities together with hematologic recovery during follow-up made this diagnosis less likely. Instead, persistent hypogammaglobulinemia with profound CD4+ T-cell lymphopenia (188 cells/µL) strongly supported an underlying combined IEI, consistent with current diagnostic recommendations [4,8].

The patient's findings were most consistent with the late-onset combined immunodeficiency (LOCID) phenotype rather than classical common variable immunodeficiency (CVID). Although LOCID is not recognized as a distinct disease entity within the current IUIS classification, it remains a clinically useful phenotype describing patients with CVID who develop profound CD4+ T-cell deficiency and opportunistic infections [1,2]. Malphettes et al. first characterized this phenotype by the coexistence of hypogammaglobulinemia, opportunistic infections, and CD4+ T-cell counts typically below 200 cells/µL [5]. Our patient demonstrated each of these defining features, including cryptococcal meningitis, recurrent herpes zoster, Ramsay Hunt syndrome, hypogammaglobulinemia, and a CD4+ T-cell count of 188 cells/µL. Nevertheless, because universally accepted diagnostic criteria are lacking, classification should rely on the overall clinico-immunologic profile rather than on any isolated laboratory or genetic finding, as emphasized by Cunningham-Rundles [6].

Our patient's prolonged diagnostic delay mirrors observations from the largest CVID cohorts, in which delayed recognition is associated with cumulative organ damage, recurrent hospitalizations, and reduced survival [9,10]. In this case, almost 20 years elapsed before the underlying immune disorder was recognized, allowing progressive immune dysfunction to culminate in life-threatening cryptococcal meningitis.

Management combined appropriate antifungal therapy with treatment of the underlying immune disorder. Resolution of cryptococcal meningitis was primarily attributable to induction therapy with liposomal amphotericin B, followed by fluconazole consolidation, whereas intravenous immunoglobulin replacement and filgrastim were initiated to correct underlying immune and hematologic abnormalities. Their use was associated with hematologic recovery, sustained clinical stability, and a marked reduction in infectious complications during follow-up, consistent with current recommendations for individualized management of symptomatic antibody deficiencies [6,11,12].

Interpretation of the FANCM c.1741C>T (p.Arg581Cys) variant also required caution. In accordance with American College of Medical Genetics and Genomics (ACMG) recommendations, variants of uncertain significance should not be used alone to establish a molecular diagnosis or guide therapy [13]. Likewise, the absence of a pathogenic variant does not rule out an IEI, as current sequencing often fails to yield a molecular diagnosis in many adult patients. Functional lymphocyte studies, vaccine-specific antibody responses, B-cell and natural killer-cell subsets, and serial post-treatment immunologic reassessment were unavailable, representing important limitations. Nevertheless, the diagnosis was supported by the convergence of the patient's longitudinal clinical history, immunologic abnormalities, exclusion of secondary immunodeficiency, multidisciplinary evaluation, and favorable clinical evolution.

For the practicing internist, cryptococcal meningitis in an HIV-negative adult should prompt consideration of an underlying inborn error of immunity. Recognition of recurrent or opportunistic infections as part of a unified clinical pattern may facilitate earlier immunologic evaluation, reduce diagnostic delay, and improve long-term outcomes through timely institution of targeted therapy.

## Conclusions

Late-onset combined immunodeficiency (LOCID), or a LOCID-like phenotype, should be considered in adults presenting with recurrent or opportunistic infections after secondary causes of immunodeficiency have been systematically excluded. In this case, cryptococcal meningitis served as the sentinel event that prompted a comprehensive immunologic evaluation, demonstrating combined humoral and cellular immune dysfunction most consistent with the LOCID phenotype. Although functional immunologic studies were unavailable and universally accepted diagnostic criteria for LOCID are lacking, the integration of the patient's longitudinal infectious history, immunologic abnormalities, and systematic exclusion of alternative diagnoses strongly supported classification within this phenotype. Appropriate antifungal therapy resulted in resolution of the acute infection, while subsequent immunoglobulin replacement and adjunctive immunomodulatory therapy were associated with sustained hematologic recovery and a marked reduction in serious infectious complications during 12 months of follow-up. This case underscores the importance of early referral to a clinical immunology service for adults with recurrent or opportunistic infections, as timely recognition of underlying inborn errors of immunity may reduce diagnostic delay, facilitate individualized management, and improve long-term clinical outcomes.
